# Recent advances in the construction of biocomposites based on fungal mycelia

**DOI:** 10.3389/fbioe.2022.1067869

**Published:** 2022-11-17

**Authors:** Ke Li, Jianyao Jia, Na Wu, Qing Xu

**Affiliations:** School of Food Science and Pharmaceutical Engineering, Nanjing Normal University, Nanjing, China

**Keywords:** biocomposites, degradable, fungal mycelium, packing material, heavy metal adsorption

## Abstract

In recent years, environmental problems have become increasingly serious, significantly effecting the ecosystem and human health. To deal with the problem of environmental pollution in an eco-conscious way, sustainable composite biomaterials are being produced. Mycelium-based composite biomaterials combine biological systems with substrates such as nanomaterials or agricultural and industrial wastes, which can complement each other’s advantages or turn waste into a useful resource. Such materials can solve practical wastewater problems as well as replace plastic products, thus reducing plastic pollution and contributing to the green transition of the environment. In this review, we summarized the recent findings of studies on these materials, indicating future research directions.

## 1 Introduction

With the rapid development of industrial technology and market economy, problems related to environmental pollution are becoming increasingly serious. This includes the “three wastes”: pollution caused by industrial production, soil pollution caused by agricultural production, and plastic pollution caused by urban activities (Shah et al., 2008; Qiu et al., 2022). The problem of environmental pollution caused by industrial production and living activities is an urgent and persistent concern worldwide as it directly harms the ecosystem and indirectly affects the quality of human life.

As most plastic packaging materials are non-biodegradable and unsustainable, causing environmental pollution when they are landfilled, the development biodegradable or composite-stable materials is being increasingly researched (Su et al., 2022). The market demand for green environmental protection materials is increasing as well. To date, a series of degradable materials have been synthesized and developed based on different biomass feedstocks, such as cellulose (Huber et al., 2011), starch (Wang B. et al., 2022), and chitin (Duan et al., 2018).

The development of bio-based materials based on these substrates has slowed down the environmental pollution caused using traditional petroleum-based materials. However, the growth and development of these substrates requires water and land, which results in a waste of resources to a certain extent; thus, it is not recommended for frequent application (Liu et al., 2020). On the other hand, fungal mycelia, which are easy to culture, grow rapidly, and have low resource requirements (Tudryn et al., 2018), are a sustainable source of multifunctional and easily degradable biocomposites which can replace traditional petroleum-based materials or other bio-based materials (Antinori et al., 2020). Therefore, the demand for these sustainable materials with low environmental impact is increasing. Recently, researchers have begun to study and develop natural biodegradable materials based on fungal mycelia with the aim of using environmental resources for green environmental protection and progressive development (Hori et al., 2013; Hyde et al., 2019).

Fungal mycelia are natural and renewable valuable structural polymer materials (Gandia et al., 2021) mainly composed of glucan, proteins, chitin, and other natural polymers (Meyer et al., 2020). Unlike bacteria and yeast, fungi, as filamentous species, can produce single tubular hyphae and to form a large-area fibrous hyphal network structure in an appropriate medium. Their hyphae can spontaneously grow entwined and colonize the substrate, which is why they are called “bio adhesives” (Meyer et al., 2011). The term “mycelium-based composite material” refers to any new degradable material formed by combining and optimizing mycelium with materials of different properties through process design. This material can be used for packaging materials, building materials, environmental repair, wearable materials. etc. (Zhang et al., 2022). Mycelium composites have the advantages of being degradable, reusable, functionally diverse, safe, efficient, and environmentally friendly (Yang et al., 2021).

**FIGURE 1:**
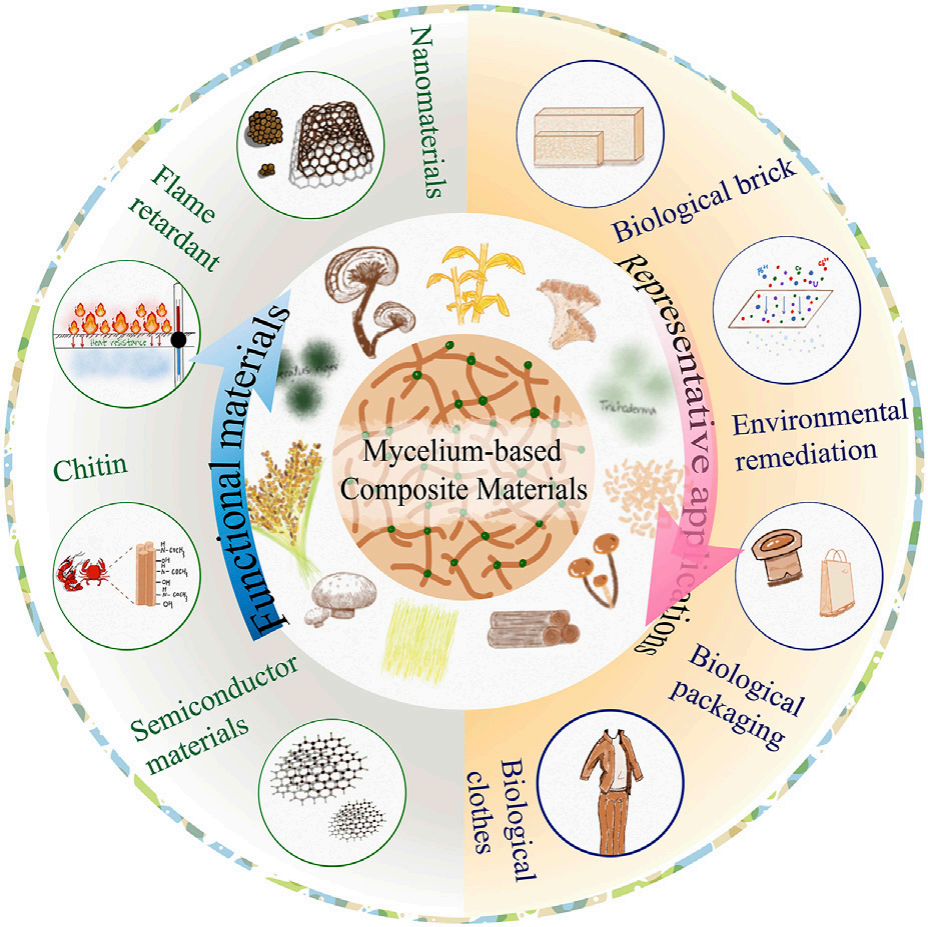
Comprehensive overview of mycelium-based composites (composite functional elements made with biomaterials to improve their biological properties and functionality).

Previous reviews (Appels et al., 2019; Girometta et al., 2019; Sharma and Sumbria, 2022) on this topic have mainly described hyphal composites based on polymer-based materials, focusing on a single function of the material. However, most reviews have not provided comprehensive descriptions of existing materials. In this review, mycelium-based composite materials were divided into two categories according to the substrates: one category includes polymer-based composites, such as industrial and agricultural wastes, while the other includes inorganic-based composite materials, such as nano-metals and semiconductors. Biodegradable materials with different functions were reviewed to improve our understanding of these materials as well as to promote their research and development (Figure 1). Continuously degradable biocomposite materials have been the focus of research in recent years and are viewed as novel cutting-edge materials.

## 2 Synthetic materials for constructing biohybrids

### 2.1 Polymers

Polymers are a class of macromolecules that can be synthesized from a wide range of natural and synthetic monomers (such as wood chips, cottonseed husks, and flax). Polymeric materials can be designed with different tunable functionalities and physicochemical properties, including mechanical, thermal, acoustic, and water absorption properties, as well as termite resistance. This has attracted diverse interest in their applications in energy, environment, and biomedicine. So far, polymers have been the most exploited material for constructing biohybrids and manipulating the biological properties of the mycelium (Alemu et al., 2022).

#### 2.1.1 Packaging materials

**FIGURE 2:**
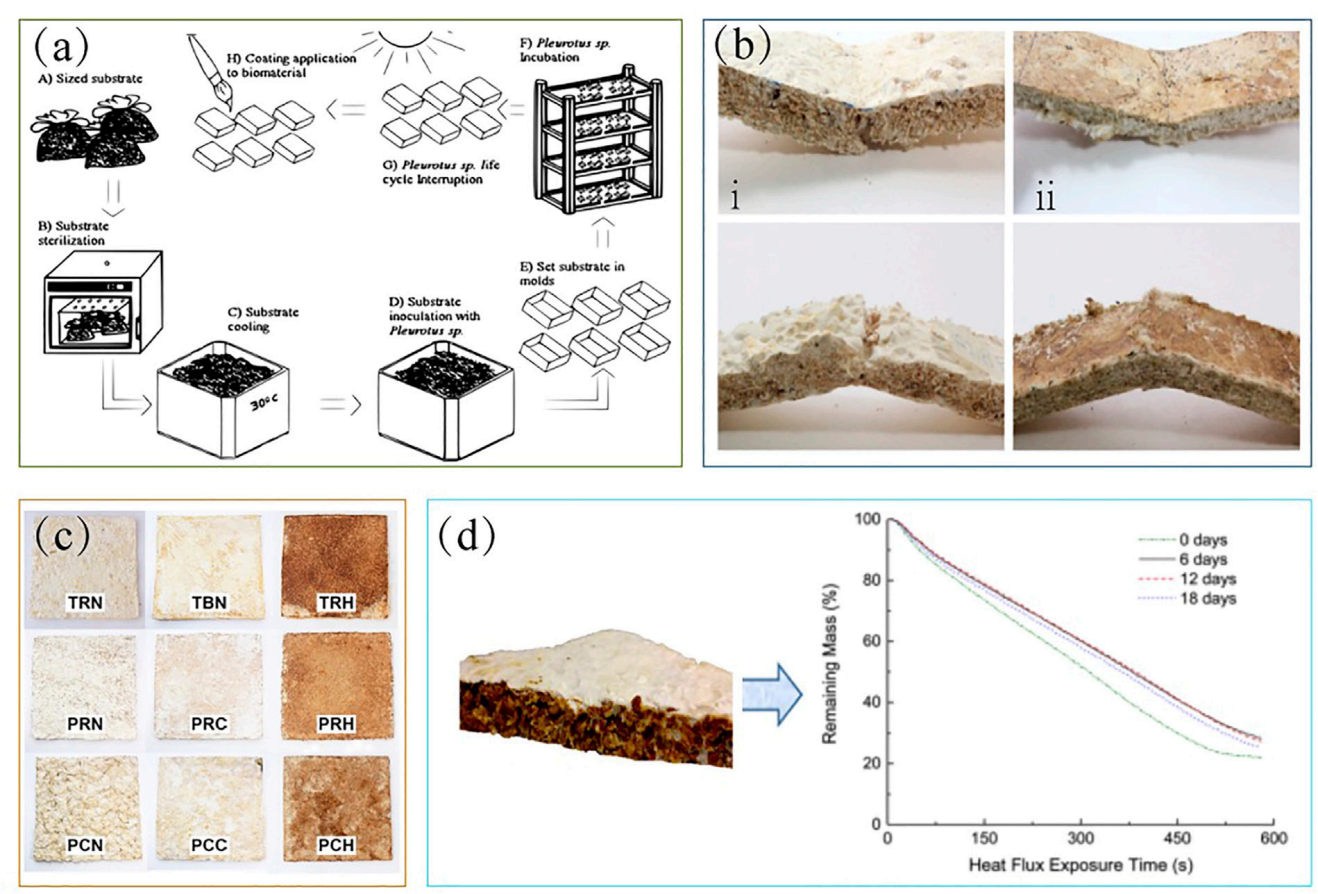
**(A)** EPS-like biodegradable composite biomaterial (López Nava et al., 2015) **(B)** comparison of different fungal materials (Elsacker et al., 2020) **(C)** comparison of different substrate matrix materials (Attias et al., 2020) **(D)** mycelium-based composite board and its thermal resistance test (Jones et al., 2018).

A varieties of researches suggest that switching to sustainable packaging could obtain a lot of benefits. Therefore the material uses the power of fungal mycelium to create an alternative to conventional packaging. Mechanical properties determine the durability of mycelium-based composite industrial products. For example, the degree of resistance depends on tensile strength, compressive strength, or elastic deformation, bending strength etc.G. A. Holt was the first to process and compound the mycelium of *Ganoderma lucidum* and cotton plant material (CPM) into cotton-based mycelium packaging materials (Holt et al., 2012). Cotton plant materials include starch and gypsum, as well as cotton cores and cottonseed hulls obtained from cotton mill waste. In this paper, six different blends with the same proportion of materials and different CPM particle sizes were tested. The fungal spores were inoculated on the mixture by two methods, namely grain and liquid culture, and the physical and mechanical properties of the products were evaluated after factory processing. The results showed that this material meets and exceeds the properties of polystyrene foam and can be used to make biodegradable packaging materials. This article was based on the research process of Bayer and McIntyre et al. J. A. López Nava et al. who modified a processing technique (López Nava et al., 2015) and composed bio-based materials using wheat crop residues and fungi oyster mushroom, *Pleurotus* sp. First, they used wheat residue particles as the matrix and oyster mushroom particles as the carrier, which were inoculated into a wooden mold for 1 month. The final product was taken out, dried, and coated with edible film (composed of carrageenan, chitosan, and xanthan gum) on the outer layer (Figure 2A). The physical and mechanical properties of the material were evaluated by applying different film types. Collectively, these results of tests showed that the composite had better tensile strength (42 kPa) than expanded polystyrene (EPS) (35 kPa), but the degree of bending (4.6–17.9 kPa)was not as good as the material created by Holt et al. (7–26.1 kPa). Therefore, the composite material developed in this study is suitable for purposes that do not require bending resistance, such as production of food packaging, small flowerpots, *etc.*

With the diversification of biomaterial mixtures and optimization of processing techniques, the mechanical and physical properties of these products can be further improved, making agricultural waste composites suitable for numerous applications using fossil fuel-based materials. The mechanical properties can be explored from three aspects: fungal type, culture substrate and processing technology.

First, the fungal type and its mycelial thickness affect the mechanical properties of mycelial composites. Lars De Laet et al. cultivated different fungi on different substrates (Elsacker et al., 2020) and showed that the compressive properties of *Trametes multicolor* (*T. multicolor*) were better than that of *Pleurotus ostreatus* (*P. ostreatus*) Han A.B.Wösten et al. used *P. ostreatus* and *T. multicolor* compounded with cotton, sawdust, and straw, and comprehensively characterized their properties after cold pressing, hot pressing, and no pressing (Appels et al., 2019). They concluded that hot pressing could change the tensile strength of composites as well as their performance, increasing the uniformity and strength of mycelium-based material, which can make it similar in performance to natural materials. At the same time, they showed that the thickness of *T. multicolor* mycelium was thicker than that of *P. ostreatus* mycelium, as well as that straw-based material was harder and less moisture-resistant than cotton-based mycelium composite material (Figure 2B).

Second, the nutrient conditions of the substrate matrix have a certain influence on the growth of fungi. In 2017, Linda S. Schadler et al. used lignocellulosic agricultural waste as the substrate matrix to explore the effect of nutrition on the mechanical properties and modulus strength of materials using two methods: inoculation and after homogenization. They found that nutritional supplementation during inoculation had little effect on the overall strength of the material, but the addition of nutrients after homogenization and mixing could improve the overall uniformity and strength modulus of the substrate matrix, which was a consequence of the formation of a continuous network of hyphae. *Regina* Helena Marino used coconut flour combined with wheat bran to promote mycelial growth, and the mycelium-based composite biomaterials colonized by *Lentinula edodes* (*L. edodes*) strains exhibited better mechanical properties when colonized for 1 month than when colonized for half a month (Matos et al., 2019). Furthermore, Andrea Ehrmann investigated whether a modified PAN nanofiber mat can promote the growth of fungal hyphae (*P. ostreatus*), change their hyphal morphology, and increase their growth rate (Sabantina et al., 2019). Noam Attias selected five plant waste substrates and four fungal species to comprehensively evaluate which combination of substrates would be most suitable for future applications (Attias et al., 2020). By testing the fitness efficiency of each substrate and fungus, they found that the best performing combination was *P. ostreatus* mycelium grown on vine and apple substrates (Figure 2C).

Third, another important thing is the difference in processing technology used to control the mechanical properties of mycelial composites. Previous studies have shown that hot and cold pressing had different effects on the mechanical properties of biocomposites. During mycelium growth, some gaps can appear because of the differences in growing speed throughout the year, and compression can reduce the porosity and increase the density of the material. Cold pressing changes tensile and flexural strengths, while hot pressing achieves even greater improvements in the material’s mechanical properties. The properties of the material change as water evaporates, temperature changes, and stress decreases. High-density materials are more widely used than low-density ones. Bajwa et al. (2017) and Pelletier et al. (2017) tried to increase the pressure during the growth and composite process, while others Liu et al. (2019), Sun et al. (2019)] tried to densify composite materials by hot pressing after the material was formed.

Other similar efforts include adding composite interlayers or compounding with materials such as cellulose nanofibers to enhance the mechanical properties of materials. For example, Mehdi Tajvidi (Sun et al., 2019) developed a new hybrid panel composite based on wood, fungal mycelium, and cellulose nanofibers (CNF) and optimized its performance at 5% CNF addition, showing its potential for packaging and furniture applications.

#### 2.1.2 Insulation materials

The most basic thermal properties of polymers are thermal expansion, specific heat capacity, and thermal conductivity. Their values vary depending on polymer state and temperature, which are closely related to the processing of products and affect the application of materials. Polymers with low thermal conductivity can be used as insulation materials. The lower the thermal conductivity of the polymer, the better its thermal insulation performance. Therefore, mycelium-based polymer composites with low density and low thermal conductivity can be used as excellent thermal insulation materials. Marli Camassola et al. (Bruscato et al., 2019) developed bio-foams using *Pycnoporus sanguineus* (*P. sanguineus*) and *Lentinus velutinus* (*L. velutinus*) cultivated on wood chips and wheat bran. Comprehensive tests showed that the bio-based materials of *P. sanguineus* and *L. velutinus* have higher density and compressive strength than those of EPS, making them an appropriate substitute for sustainable insulation materials. Lars De Laet et al. processed white rot fungi and five lignocellulosic materials (hemp, flax, flax waste, cork, and straw) with five fiber conditions (loose, chopped, dust, pre-compressed, and tow) to investigate their properties and found that mycelium composites containing hemp, flax, and straw exhibited good thermal insulation properties, but not as good mechanical properties (Figure 2D). Furthermore, mycelium-based biocomposites showed a significantly reduced tendency to burn compared to those of polymethylmethacrylate (PMMA) and polylactic acid (PLA) materials, indicating that they are less prone to fire and thus safer to use (Jones et al., 2018). The mycelium-based composite biomaterials studied by Tanmay Bhat et al. were found to have good flame-retardant properties. Chitosan has been studied as a promising flame retardant additive (Hu et al., 2012), mainly relying on the substance being stable at high temperatures, with a peak value of 300°C. In addition, hydrophobin (Appels et al., 2018) and lignin (Yu et al., 2017) can also be used as natural flame-retardant additives, as they were shown to improve the flame-retardant properties of mycelium-based composites.

Acoustic performance is a physical property of sound that affects our quality of life. Mycelium-based composite materials can absorb sound by pressing the material and increasing its density. The higher the density, the better the noise reduction coefficient and sound insulation effect. M.G. Pelletier et al. (2017) grew a mycelium based on agricultural by-products such as cotton by-products, cotton burrs, straw, sorghum stems, and corn stalks, and then compressed the original products into high-density composite materials to form natural lightweight bio-composite panels that can be applied to acoustics. This mycelium absorbed 50%–70% of the sound. The results showed that the sound insulation performance gradually improved and almost reached 0.087 g/cc. Afterwards, increased density also does not enhance sound insulation. Unlike modern commercial acoustic insulation materials, this novel mycelium-based composite density material provides good sound insulation and is a promising bio-based composite alternative to sound shielding panels.

**FIGURE 3:**
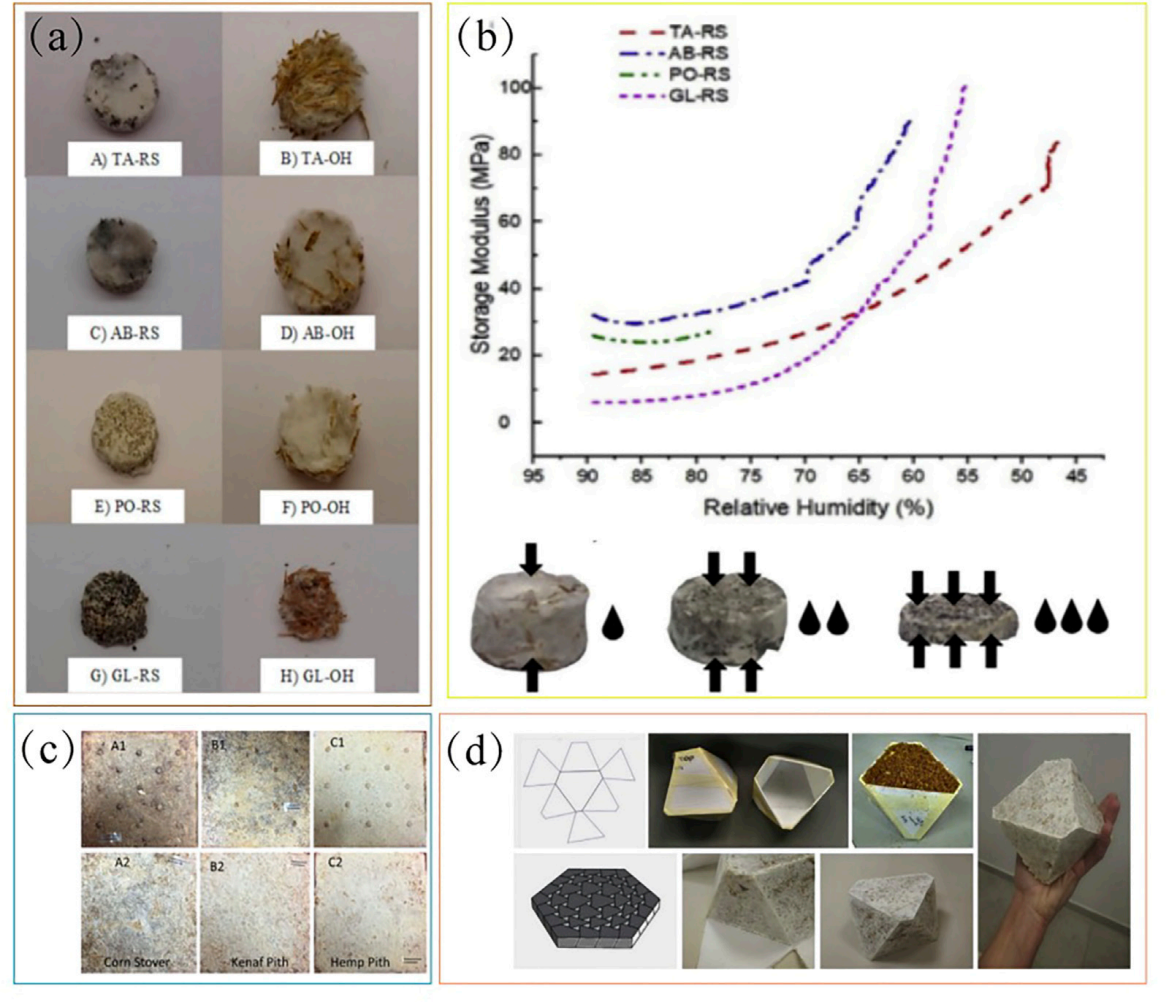
**(A)** Development of moisture-resistant composites based on different matrices (*Trichoderma asperellum* (TA) grown on A) rapeseed cake (RS) and B) oat husks (OH) as substrates; Agaricus bisporus (AB) grown on C) RS and D) OH; Pleurotus ostreatus (PO) grown on E) RS and F) OH; Ganoderma lucidum (GL) grown on G) RS and H) OH), **(B)** their dynamic moisture monitoring and schematic diagram of material changes (Tacer-Caba et al., 2020), **(C)** development of termite-resistant composite bio-boards (Bajwa et al., 2017), **(D)** specific models constructed based on CAD design and innovative molds (Attias et al., 2020).

Different fungal strains have different hydrophobic properties. The stronger the hydrophobicity, the higher its potential to be used as a moisture-resistant material. However, a common problem with mycelium-based materials is their high-water absorption capacity, which has certain restrictions on the use of their materials. The properties of these materials can be adjusted by pressing them or finding suitable strains. Kirsi S. Mikkonen used *Agaricus bisporus*, *Trichoderma asperellum*, *Pleurotus ostreatus* and *Ganoderma lucidum* and compounded them with oat mixture and rapeseed cake (Figure 3A). They obtained eight types of fungal composites after oil pressure growth (Tacer-Caba et al., 2020). In contrast to EPS (50 kg/m^3^), the density of foam-like material based on mycelia can range from 59 to 318 kg/m^3^. In the present study, the RS-fed hyphal composite (561 kg/m^3^) had a higher density than the OH-fed hyphal composite (230 kg/m^3^). Therefore, the comprehensive results showed that the products of *A. bisporus* grown on rapeseed cakes had strong hydrophobic properties and can be used to produce moisture-resistant plastic products. This was the first study to measure the dynamic moisture-retaining properties of fungal composites (Figure 3B). To sum up, the heat, sound and moisture resistance of mycelium-based materials can be improved by increasing the density.

In addition to this, mycelium-based termite resistant composites were also investigated to resistant to termite. The mycelium-based degradable composite material studied by Dilpreet S. Bajwa was composed of fungal mycelium, lignocellulose, and termites. Biocomposite panels with two densities were fabricated using three different fungal strains (*Daedaleopsis confragosa*, *Ganoderma resinaceum*, and *Tramates versicolor*) based on kenaf, hemp, and corn stover fibers (Bajwa et al., 2017). The termite resistance of these bio composite panels was evaluated using four termiticides (vetiver oil, guayule resin, cedar oil, and borax). Comprehensive tests showed that resin-treated kenaf and hemp boards had the strongest repellency to termites and could be used as effective anti-termite bio-based composites (Figure 3C).

### 2.2 Summary

Further innovations and optimizations of the materials described above include the use of plant crop waste, design, and adjustment of molds according to CAD, construction of specific shapes, and achievement of mycelium-based self-fusion growth, so that such materials can meet different requirements and promote the sustainable development of bio-based fungal materials. For example, Yasha Grobman and Michael Weizman used a laser to cut and fold into 37 unique 3D molds that can be self-assembled to form suspended ceiling models, known as topologically interlocking mycelial bricks (Attias et al., 2020) (Figure 3D). In addition to this, many studies have evaluated other properties of mycelial materials. For example. Jones et al. (2020) and. Girometta et al. (2019) comprehensively evaluated the basic and engineering properties (including antibacterial properties and shape flexibility) of mycelial bio-based materials et al. (Kuribayashi et al., 2022).

The ability to develop low-cost and sustainable environmentally friendly composite materials has attracted commercial and academic interest. Such mycelium-based composites are constructed from complex network structures of fungal mycelia grown on natural or waste substrates (Jones et al., 2017). Filamentous hyphae grow and colonize the substrate, acting as a natural gum which provides mechanical support. Substrates for growing mycelial complexes can mainly be recovered from industrial or agricultural wastes at low prices, reduce the production costs and achieving economic benefits. The materials have the characteristics of reusability, diverse functions, and easy operation. To a certain extent, they can replace petroleum-based plastic products, thus effectively reducing environmental pollution and our negative impact on the ecological environment and human health, aiming to achieve environmental protection and sustainable use of resources. Although some basic research has been carried out on such materials, at this research stage, some problems still exist:

1) Few studies on these topics have investigated the fungi used for making these composite materials, even though researching the properties of different fungal strains is necessary to find more suitable strains to produce different composite materials (Hyde et al., 2019).
2) It is necessary to develop low-cost and sustainable substrates for industrial production.
3) The composite design is not its most important property; the process production and manufacturing also need to be optimized, which can be combined with other data technology.

In addition, the appearance of these materials is still not as appealing, as they are currently in rough processing.

## 3 Inorganic materials

Inorganic materials are divided into metal and non-metal materials, mainly including nano-metal and semiconductor materials. Nano-metal materials are metals and alloys that form nano-grains. They are characterized by large specific surface area and high adsorption capacity and used as good adsorbents in combination with fungal hyphae. The currently used application substrates mainly include nano-metal oxides, magnetic nanoparticles, and nano-gold particles, which are used in wastewater treatment, as catalysts, *etc.* (Rashid et al., 2020). Inorganic non-metallic materials are materials composed of oxides, carbides, aluminates, and other substances of certain elements. Inorganic non-metallic functional materials have many functions because of their semiconductor properties, adsorption properties, and radiation resistance. The materials compounded with mycelia include semiconductors, such as graphene oxide, which are used for the absorption of radioactive substances (Zhu et al., 2019).

### 3.1 Heavy metal absorbing and catalytic materials

**FIGURE 4:**
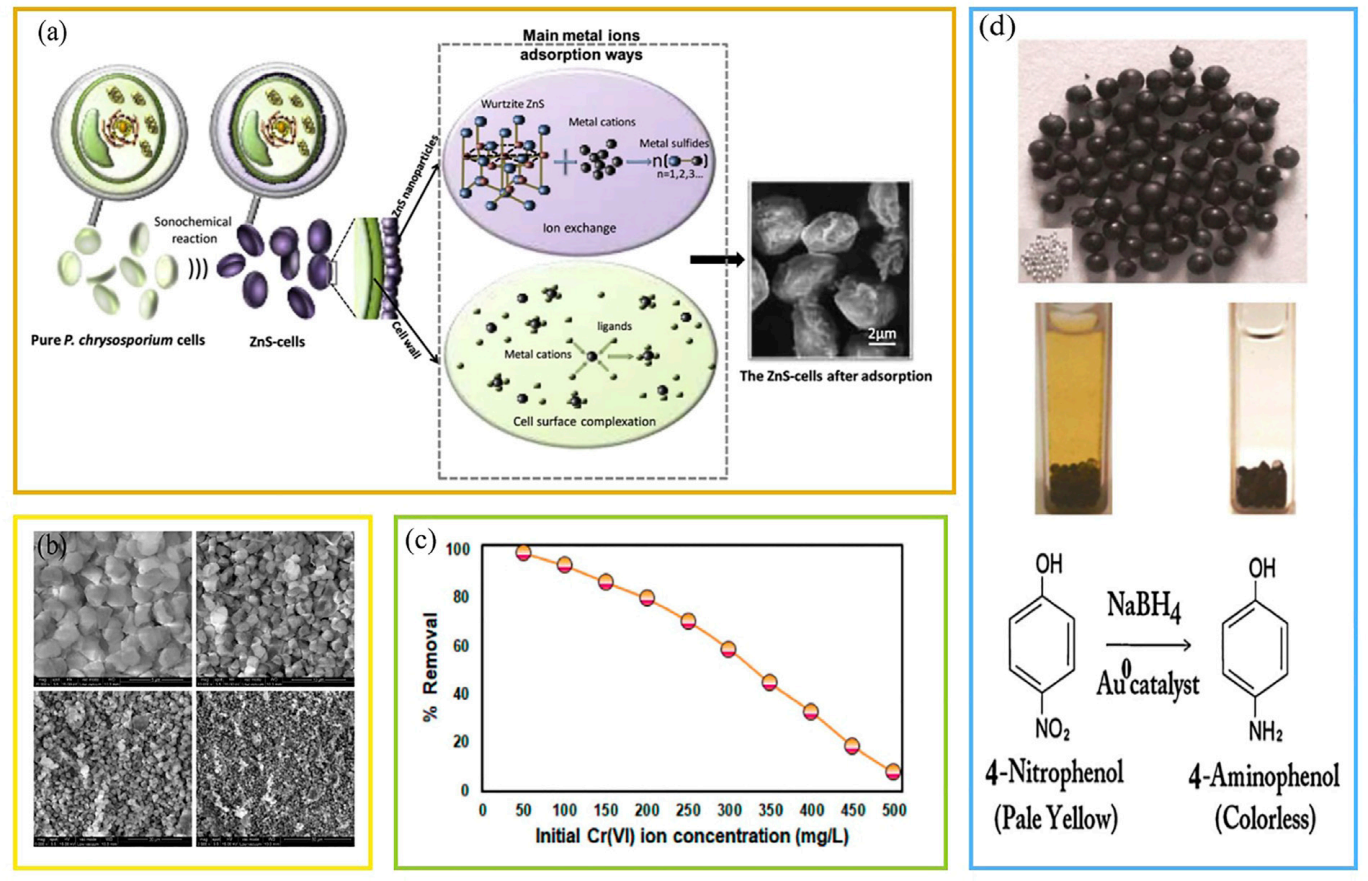
**(A)** High-performance adsorption of Pb^2+^ and Cd^2+^ by ZnS nanoparticle layer (Qin et al., 2020), **(B)** synthesis of magnetic nanoparticles coated mixed fungal biomass (MNP-FB), **(C)** adsorption of Cr(VI) ions (Saravanan et al., 2021), **(D)** nanocomposite biomass synthesis of materials and their heterogeneous catalysis to enable the recovery of AuNPs catalysts: schematic representation after catalytic adsorption (Narayanan and Sakthivel, 2011).

Nano-metal sulfides have attracted extensive attention because of their simple fabrication process, low cost, and strong ion exchange capacity. Zhou et al. (2007) used sonochemical methods to synthesize ZnS nanostructures using microorganisms as templates. Pala and Brock. (2012) used ZnS nanoparticle gels to remove Pb^2+^ and Hg^2+^ from heavy metal sewage. Jaiswal et al. (2012) showed that ZnS quantum dots-chitosan films can be used to remove heavy metal ions from wastewater. Fang et al. (2018) used ZnS nanocrystals for the sequential removal of multicomponent heavy metals in aqueous solutions. Hu et al. (Qin et al., 2020) successfully synthesized ZnS nanoparticles on the cell surface of *P. chrysosporium*(BKMF-1767) cells using a sonochemical method, obtaining a high-performance composite adsorbent. Compared with primitive fungal cells, the adsorption capacities of these developed materials for Pb^2+^ and Cd^2+^ were increased by 140% and 160%, respectively (Figure 4A). For removing Cr^6+^ from wastewater, P. Senthil Kumar used newly synthesized magnetic nanoparticles mixed with fungi (*Aspergillus niger* and *A. fumigatus*) to cultivate the MNP-FB material (Saravanan et al., 2021) (Figure 4B). The monolayer adsorption efficiency of Cr^6+^ by MNP-FB was 249.9 mg/g, making it an economically feasible material (Figure 4C). This mycelium-based composite study provided a feasible and novel method for preparing a heavy metal wastewater adsorbent by combining microorganisms and nanomaterials, which has great potential for practical wastewater treatments.

For the first time, Narayanan and Sakthivel. (2011) developed a green chemistry-stable spherical gold nanocomposite with high catalytic activity based on nano-gold material biocomposite with fungal mycelium (*Cylindrocladium floridanum*). This material can quantitatively and heterogeneously reduce 4-NP to 4-AP, reducing the environmental harm of toxic organic pollutants (Figure 4D). Heterogeneous catalysts, supported by fungal biomass, promote biocatalysis, and increase reaction rates. However, considering their economic feasibility and environmental friendliness, an in-depth understanding of sustainable green biocatalytic reduction of various nitro pollutants is required for their further use.

### 3.2 Radioactive absorbing materials

**FIGURE 5:**
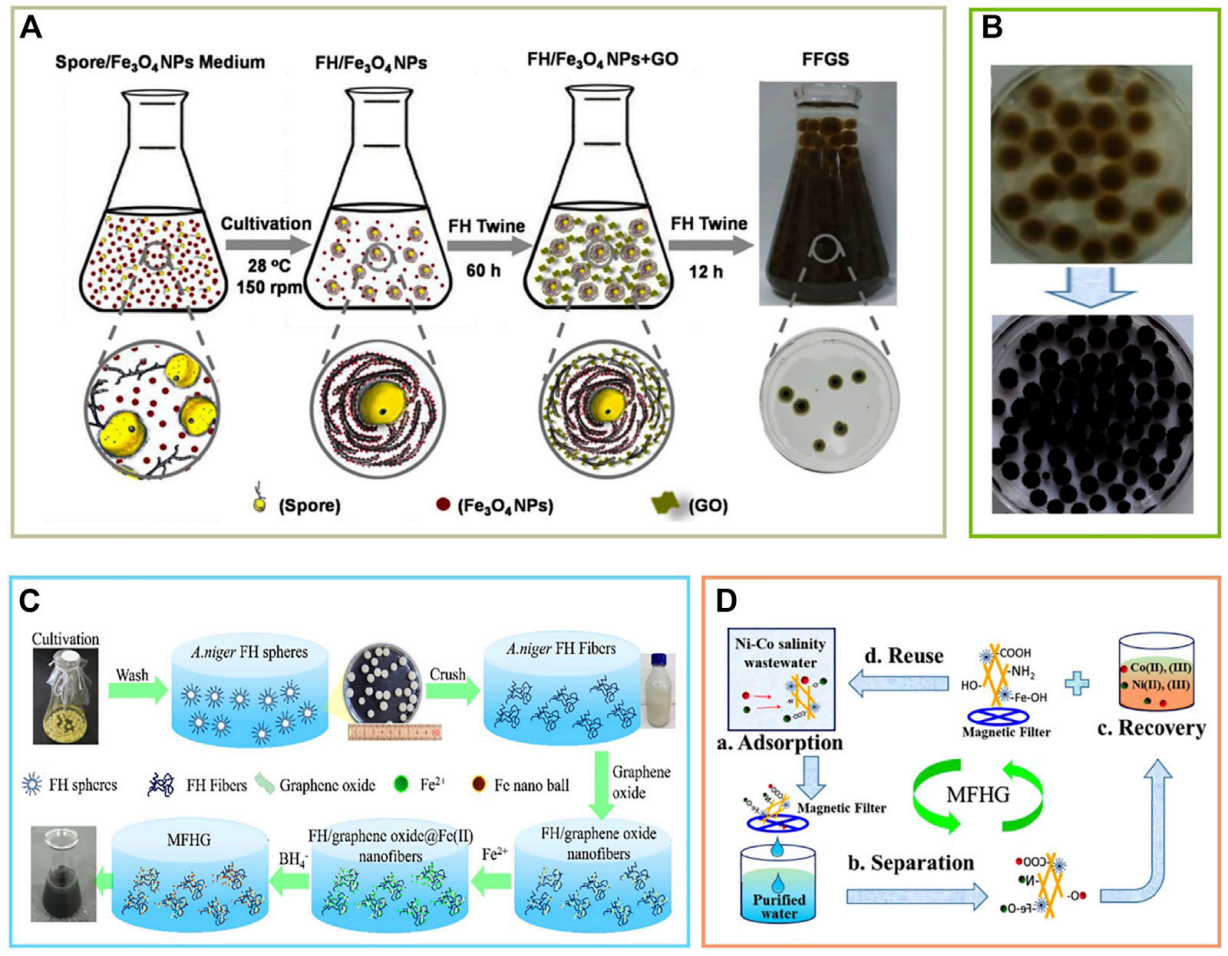
**(A)** Synthesis of layered core-shell structured fungus hyphae (FH)/Fe_3_O_4_/graphene oxide (GO) nanocomposite spheres (FFGS), **(B)** removal and recovery of methyl violet (MV) and uranium (U) (after 72 h) (Zhu et al., 2019), **(C)** preparation of three-dimensional magnetic fungal hyphal/graphene oxide nanofibers (MFHG), **(D)** capture and adsorption of metal ions Co (II) and Ni (II) (Li et al., 2019).

Graphene oxide (GO) plays an important role in wastewater treatment because of its high specific surface area and fast reaction kinetics. Zhu et al. (2019) successfully prepared hierarchical core-shell structured FH/Fe_3_O_4_/GO nanocomposite spheres (FFGS) by continuously culturing fungal hyphae (FH) in Fe_3_O_4_- or GO-containing media (Figure 5A). The bulk adsorption results showed that the composite FFGS was much better than FH, FH/GO, and FH/Fe_3_O_4_ in the adsorption of methyl violet (MV) and uranium (U), which may be attributed to its lower zeta potential (Figure 5B). Therefore, the core-shell structured FFGS is a promising adsorbent for the removal and recovery of MV or U(VI) from wastewater, and this strategy is low-cost and environmentally friendly. Nanosheets based on graphene oxide have also been developed. He et al. (Chen et al., 2021) used photodegradation to develop FH-graphene-MoS_2_ hybrid nanosheets, which can effectively adsorb radioactive U ions. However, this nanomaterial has a low recovery rate and poor stability, which should also be considered in future research. Wang et al. (Li et al., 2019) assembled three-dimensional magnetic fungal hyphae/graphene oxide nanofibers (MFHG) using a self-assembly reduction (RSA) strategy for the purpose of efficient capture of Co and Ni from high-salinity aqueous solutions, improving the stability of the material (Figures 5C,D). Based on MFHG, a continuous recovery reactor (CFRR) for treating aqueous solutions was developed, which exhibited high efficiency in removal and regeneration. The combination of MFHG and CFRR is a promising and efficient method for wastewater treatment. For heavy metal adsorption and radioactive material absorption, the adsorption effect of mycelium-based materials can be judged by fitting adsorption kinetics.

Inorganic materials, including nanoparticles and semiconductors, have the advantages of strong self-assembly ability, high specificity, and good reactivity, which make them the preferred substrates for the treatment of water pollution and other harmful pollutants. Therefore, adsorbents formed by the composite of inorganic and biological materials can rapidly adsorb heavy metals and radioactive substances in wastewater. These materials can not only make up for the weak mechanical properties and limited adsorption capacity of microorganisms but can also neutralize the shortcomings of the difficult separation of inorganic materials, resulting in a significant increase in wastewater adsorption efficiency (Holkar et al., 2016).

The functional materials developed by composing such inorganic materials and fungal mycelia are promising treatment materials for the adsorption of harmful substances (Rashid et al., 2021). However, such mycelium-based composite functional materials are rarely studied and are currently only used in laboratories. Thus, more research on their industrial adaptability is needed to achieve the goal of low cost and high adsorption.

## 4 Concluding remarks and future perspectives

In the context of environmental pollution, this review discusses and summarizes the advantages of composite biomaterials, focusing on mycelium materials. Mycelium-based composite biomaterials can be used as packaging materials (Nawawi et al., 2020), building decoration materials, environmental restoration materials (Holkar et al., 2016), and for many other purposes. With the progress of research, the genome sequences of many species have been revealed, allowing them to be genetically modified. The research on mycelium-based composite biomaterials is still at the beginning, and it is a popular future research direction. With the crossover of multiple disciplines and diversification of research, the development of composite biomaterials progresses from ordinary simple compounding to gene editing synthesis, that is, synthetic biology is being used to transform mycelium-based composites and utilize chassis cells in biological systems to endow them with new functions (Wang Y. et al., 2022). By designing, transforming, and recombining biological cell systems, and then combining them with new functional elements, we can develop many efficient, safe, and environmentally friendly products for many purposes. Because of their diverse biological applications and advanced technical means, composite biomaterials have good application prospects in the fields of bioenergy, environmental improvement, medicine, food technology, and health care, and can thus provide many benefits for human social development and ecological health. Although there are still many problems that need to be addressed in the currently available mycelium-based composite materials, as cheap and degradable materials, their researching, and commercialization trend is increasing day by day. Their main advantages are their broad application potential and market prospects, and their potential in promoting sustainable development in the future.
